# The Adulteration of Food

**Published:** 1869-01

**Authors:** 


*The Adulteration of Food.*—The extent to which the adulteration of the various articles of food is now carried on is beginning to attract considerable attention. Recent investigations made in New York City, show that the loss from this cause and that of false weights, to be as great as fifteen per cent. The following account from the reporter of the *World*, who made extensive purchases of articles which were afterwards reweighed and submitted to a chemical analysis will show the manner in which fraud is thus perpetrated:

"Among the articles adulterated, coffee is prominent, it being the habit of fraudulent dealers in New York, as in England, to mix it largely with chicory and also ground beans. Brown sugar is tampered with by the use of flour, potatoes and tapioca starch; even sand and plaster are sometimes introduced to make bulk and weight, and, aside from the adulterations, some of the lower grades of sugar are frequently filled with immense numbers of live animalculæ, said to be the cause of the disease known as the grocer's itch. Vinegar is often little less than diluted snlphuric acid largely adulterated with arsenic; even corrosive sublimate is used by some manufacturers to give strength to vinegar, and pickels are often boiled in copper to give them the light green color which is so much admired. An old trick of swindling flour-dealers is to open the bottom of a barrel of fine flour and substitute an article of inferior quality. When the buyer opens the top of the barrel he finds it all right, but when he gets about half way down he discovers the fraud by which the grocer has gained from one to two dollars. False weights are alleged to be practiced not only at groceries of New York, but the markets and the coalyards. They prevail most in the article of tea, which is adulterated besides, being a compound of "tea leaves once used, sloe, oak, willow, elm, poplar and hawthorn leaves, with various poisonous colorings, and catechu and sulphate of iron for taste."

The common candies and confectionaries of commerce are far from being wholesome, if recent investigations are to be believed.

"The adulteration of sugar candies and spices is a trade largely and regularly carried on in this city. Instead of plaster which till lately entered so largely into the manufacture of confectionary, in place of sugar, a new article has been discovered called *terra alba*, or white earth. It comes from Ireland, and costs by the barrel about 1½ cents per pound, while loaf sugar costs about 17 cents.

The bodies of candies, the coating of almonds and lozenges, are made from this earthy material. It is whiter than plaster, and is very much used in the adulteration of flour sold in this market. A glue, paint and oil manufacturer of New York has sent round his annual circular, which I have seen, to the principal confectioners, calling attention to a fresh arrival of this white earth. I have seen an ounce of lozenges dissolved in water, in which two-thirds of an ounce was terra alba, and not a particle of sugar in the lot.

The common method of flavoring candies, almonds, sugar-plums, etc., is with deleterious substances. The pine-apple flavor, the banana and the peach, are made from fusil oils, which are very poisonous. Bitter almond flavor is made from prussic acid undadulterated. Pine-apple flavor is obtained from rotten cheese, very rotten, and nitric acid.

Gum arabic for pure gum drops is costly. An article has been invented of the most beautiful appearance, that is used instead of the gum. It is very cheap and very poisonous. In pure candy, cochineal is used to color red, and saffron for the yellow. But in common candies poisonous coloring is put, the same that is used to color wines and liquors. One of the most common is "carlot" into which arsenic largely enters. A few grains of this substance will color a cask of wine. Liquorice drops for the "trade" are made of poor brown sugar, glue and lamp-black, flavored with liquorice. And for the Western trade much of this vile stuff is packed, and sent West to be put up in boxes to suit the market, of which from 75 to 90 per cent is terra alba. This material enters largely into the common chocolates and spices. Much of the cream of tartar used for bread is made of terra alba and tartaric acid."

